# Immune Trajectories After Switch to Long-Acting Cabotegravir Plus Rilpivirine in the RELATIVITY Cohort

**DOI:** 10.1093/ofid/ofag583

**Published:** 2026-10-23

**Authors:** Jesús Troya, Noemí Cabello-Clotet, José Ignacio Bernardino, María José Galindo, Rosa María Martínez, Juan Martín, María Remedios Alemán, Miguel Torralba, Francisco Fanjul, María Carmen Fariñas, Adrián Rodríguez, María José Crusells, Sonia Calzado, Javier Pérez-Serrano, Carmen Hidalgo-Tenorio, Luis Enrique Morano, David Vinuesa, Enrique Bernal-Morell, Inmaculada Poquet-Catalá, Hadrián Pernas-Pardavila, Juan Tiraboschi, Diana Corps, María Jesús Vivancos, Luis Buzón-Martín, RELATIVITY Cohort, Ana Cristina Antolí Royo, Carmen Grande Sáez, María Ángeles Garcinuño Jiménez, Ana Carrero Gras, Eva María Ferreira Pasos, Hadrian Pernas Pardavila, Mercedes González, Nuria Vázquez Temprano, Miriam Estébanez, Tatiana Mata-Forte, Ana Muñoz Gómez, Claudia Dorta Hernández, José Reynaldo Homen Fernández, Juncal Pérez-Somarriba Moreno, Maravillas Carralón, María José Núñez Orantos, María Nieves Sanz Pérez, Noemí Cabello-Clotet, Philip Erick Wickman Jorgensen, Susana Olmedo Hernández, Teresa Martínez Burgoa, Valeria Cabral Sousa, Vicente Estrada Pérez, Virginia Víctor Palomares, Ana Ferrer Ribera, Andreu Belmonte, Ángela Pradilla Dieste, Carolina Pinto, María José Galindo Puerto, Rosa Oltra Sempere, Sandra Pérez Gómez, Álvaro Cecilio, Isabel Sanjoaquín Conde, María José Crusells Canales, Pilar Jiménez Marcen, Ana Lucas Dato, Belén Martínez López, Inmaculada González Cuello, Jara Llenas García, María García López, Carolina Navarro, Luis Buzón Martín, María Fernández, Amparo Exojo Morales, Carlos de Andrés David, Inmaculada Poquet Catalá, Karenina Antelo Cuéllar, Laia Navarro Peiró, María Josefa de la Asunción Villaverde, Silvia Lope Bolumar, Alba León Barbosa, Esther Nogales Nieves, Eva García Alcalde, Juan José Corte García, Lucía Alonso Alonso, María Vanessa López Peláez, Sara Jaber Carballo, Albert Gómez Lozano, Sara García Torras, Marta Milián Sanz, Ana Isabel Lérida Urteaga, Antonia Pérez Barja, Javier López-Nieto, Magdalena Muelas Fernández, Beatriz de la Calle, Ángela Botella Zaragoza, Félix Gutiérrez Rodero, Javier García Abellán, Mar Masiá, Paula Mascarell Arlandis, Sergio Padilla Urrea, Chiara Fanciulli, Cristina Díez Romero, Francisco Tejerina Picado, Juan Carlos de Quirós López Bernaldo, Leire Pérez Latorre, María Teresa Aldamiz-Echevarría Lois, Antonio Jesús Sánchez Guirao, Ángeles Muñoz Pérez, Antonia Alcaraz García, Cristina Tomás Jiménez, Elena Guijarro Westermeyer, Enrique Bernal Morell, Eva García Villalba, Joaquín Bravo Urbieta, María Dolores Hernández Lorente, Rodrigo Martínez Rodríguez, Román González Hipólito, Noemí Ramos Vicente, Patricia Barragán Gallo, Alberto Juárez Toquero, Beatriz Valentín Casado, Jesica Abadía Otero, Julia Gómez Barquero, Pablo Bachiller Luque, Yolanda Arranz García, Adriana Pinto, David Rial, Federico Pulido, Juan Martín, Laura Bermejo Plaza, María de Lagarde, María Teresa López Caballero, Mireia Santacreu, Otilia Bisbal, Rafael Rubio, Roser Navarro, Victoria Macheño del Real, Celia Miralles Álvarez, Guillermo Pousada, Henar de las Heras Miralles, Luis Enrique Morano Amado, Víctor Asensi, Laura Guio Carrión, Mikel del Álamo, Ester Sáez de Adana Arroniz, Juan Carlos Gainzarain Arana, Ana Vegas Serrano, Juan Emilio Losa García, María Velasco Arribas, Maite Ganchegui Aguirre, Miriam López Martínez, Óscar Luis Ferrero Benítez, Alicia Sedo Mor, Analuz Fernández, Juan Tiraboschi, Josefa Soler González, Víctor Arenas García, Lucía Romero, Nereyda Tosco García, Remedios Alemán Valls, Guillermo Soria Fernández-Llamazares, Juan Víctor San Martín López, Ruth Calderón Hernáiz, Alberto Delgado Fernández, Lorenzo Sánchez, Miguel Torralba González de Suso, Alberto Romero Palacios, María Antonia Sepúlveda, Alejandra Gimeno García, Alejandro David Bendala Estrada, Carmen Montero Hernández, Diana Corps Fernández, Jose Carlos Escribano, María del Mar García Navarro, Marouane Menchi Elanzi, Ana Cerezales Calviño, Bárbara Alonso Moreno, José Fernando Lluch Perales, Alfonso Cabello, Miguel Górgolas Hernández-Mora, María Rubio Olivera, Marta Clavero Olmos, Belén Escribano Losada, Guillermo Cuevas, Jesús Troya García, Mariano Matarranz, Pablo Ryan, Sandra Rodríguez Perut, Alejandro de Gea, Ana Belén Delgado Hierro, Carmen Busca, Jose Ignacio Bernardino, Juan González García, Luz Martín Carbonero, María del Mar Arcos Rueda, María Luisa Montes, Rafael Micán, Rocío Montejano, Andoni Casen Gil, Ignacio de los Santos, María Aguilera García, Aitziber Illaro Uranga, Carlos Armiñanzas Castillo, Claudia González-Rico, Francisco Arnaiz de las Revillas Almajano, Manuel Gutiérrez Cuadra, María Carmen Fariñas Álvarez, Noelia Ruiz-Alonso, Paula Runza, Raúl Parra-Fariñas, Ángela Forcén Vicente de Vera, María Aranzazú Caudevilla Martínez, Rosa María Martínez Álvarez, Ruth Caballero Asensio, David Dalmau Juanola, Laura Gisbert Pérez, Mireia Cairó Llobell, Roser Font Canals, Xavier Martínez Lacsa, José Sanz, Alberto Díaz de Santiago, Ana Fernández Cruz, Sara de la Fuente Moral, Ana Moreno, Daniel de las Heras Gómez, María Jesús Pérez Elías, María Jesús Vivancos Gallego, Mario Pons Guillén, Desiré Pérez Martínez, María Elisa Pino Díaz, Miguel Alberto de Zárraga Fernández, Miguel Vicente Egido Murciano, Teresa Omiste Sanvicente, Adrián Ferré, Antoni Campins, Francisco Fanjul, Luisa Martín Pena, Melchor Riera, Adrián Rodríguez, Patricia Sorní Moreno, Carmen Hidalgo Tenorio, Sergio Sequera Arquelladas, Cristina Escrich, María del Carmen Navarro, David Vinuesa

**Affiliations:** Hospital Universitario Infanta Leonor, Madrid, Spain; Hospital Universitario Clínico San Carlos, Madrid, Spain; Hospital Universitario La Paz, Madrid, Spain; Hospital Clínico Universitario de Valencia, Valencia, Spain; Hospital Universitario Miguel Servet, Zaragoza, Spain; Hospital Universitario 12 de Octubre, Madrid, Spain; Hospital Universitario de Canarias, Santa Cruz de Tenerife, Spain; Hospital Universitario de Guadalajara, Guadalajara, Spain; Hospital Universitari Son Espases, Palma de Mallorca, Spain; Hospital Universitario Marqués de Valdecilla, Santander, Spain; Hospital Universitario Son Llatzer, Palma de Mallorca, Spain; Hospital Clínico Universitario Lozano Blesa, Zaragoza, Spain; Consorci Corporació Sanitaria Parc Taulí, Sabadell, Barcelona, Spain; Medicina Interna. Hospital Universitario La Princesa, Madrid, Spain; Hospital Universitario Virgen de las Nieves, Granada, Spain; Hospital Universitario Álvaro Cunqueiro, Vigo, Pontevedra, Spain; Hospital Universitario Clínico San Cecilio, Granada, Spain; Hospital General Universitario Reina Sofía, Murcia, Spain; Hospital Universitario de Denia-Marina Salud, Alicante, Spain; Complexo Hospitalario Universitario de Pontevedra, Pontevedra, Spain; Hospital Universitario de Bellvitge, Barcelona, Spain; Hospital Universitario de Torrejón, Madrid, Spain; Hospital Universitario Ramón y Cajal, Madrid, Spain; Hospital Universitario de Burgos, Burgos, Spain

**Keywords:** cabotegravir plus rilpivirine, CD4/CD8 ratio, CD4+ and CD8+ T cells, long-acting injectable, real-world data

## Abstract

**Background:**

Immune and virological trajectories after switching to long-acting cabotegravir plus rilpivirine (CAB+RPV LA) remain incompletely characterized in routine care settings.

**Methods:**

We analyzed virologically suppressed adults switching to CAB+RPV LA in the Spanish multicenter RELATIVITY cohort. Paired CD4+, CD8+, and CD4/CD8 measurements were assessed at approximately 12 and 24 months and at the last available immune assessment. Analyses used a 1.0 ratio threshold and incorporated longitudinal HIV-1 RNA, recorded geographic origin, pre-switch suppression duration, and immediate pre-switch antiretroviral therapy.

**Results:**

Among 3349 participants, the last paired-ratio assessment (n = 2929) occurred at a median of 22 months (IQR, 16–27). Median changes were −7 cells/mm^3^ (IQR, −138 to +117; n = 2992) for CD4+ T-cell count, −60 cells/mm^3^ (IQR, −214 to +75; n = 2941; *P* < .001) for CD8+ T-cell count, and +0.07 (IQR, −0.05 to +0.20; n = 2929; *P* < .001) for the CD4/CD8 ratio. Baseline CD4/CD8 <1.0 was independently associated with greater ratio increase (adjusted beta, +0.060; 95% CI, +.030 to +.090; *P* < .001). Longer pre-switch suppression and any post-switch HIV-1 RNA ≥50 copies/mL were associated with smaller ratio increases. Adjudicated virological failure occurred in 28/3349 (0.8%). Findings persisted among participants with all recorded post-switch HIV-1 RNA values <50 copies/mL and after excluding protocol-defined virological failure.

**Conclusions:**

During effective CAB+RPV LA maintenance, CD4+ T-cell counts showed no meaningful change, while a modest decline in CD8+ T-cell counts was accompanied by a small CD4/CD8 ratio increase. These descriptive changes are compatible with continued immune evolution during sustained suppression and do not establish a CAB+RPV-specific effect or justify additional routine T-cell-subset monitoring.

## Teaser

Among 3349 individuals with HIV who switched to long-acting cabotegravir plus rilpivirine, CD4+ T-cell counts remained stable, while a modest increase in the CD4/CD8 ratio was driven by a decline in CD8+ T-cell counts; ratio increases were smaller among those with longer prior virological suppression and those with post-switch viremia during follow-up.

Contemporary HIV-1 care increasingly focuses on outcomes beyond durable virological suppression. Effective antiretroviral therapy (ART) restores CD4+ T-cell counts in most people with HIV (PWH); however, expanded CD8+ T-cell populations, a low CD4/CD8 ratio, and residual inflammation may persist for years [1–8]. Absolute CD8+ T-cell count and the CD4/CD8 ratio have been associated with immune senescence, non-AIDS morbidity, and mortality, although their incremental prognostic value over conventional clinical and virological measures remains debated [4–11].

Long-acting cabotegravir plus rilpivirine (CAB+RPV LA) replaces daily oral ART with scheduled intramuscular injections. Randomized trials and real-world cohorts have demonstrated high virological efficacy and durability [12–19]. CD4+ and CD8+ T-cell trajectories, however, have seldom been followed longitudinally after a transition to this strategy.

Immune trajectories during suppressive ART may reflect baseline immune status, duration of prior virological suppression, and subsequent episodes of detectable viremia, rather than the maintenance regimen alone [5–11]. Demographic and clinical factors, including age, sex, recorded geographic origin, comorbidity burden, and treatment history, may also influence the magnitude of these changes.

Using a nationwide Spanish RELATIVITY cohort, we characterized paired changes in CD4+ and CD8+ T-cell counts and the CD4/CD8 ratio after switching to CAB+RPV LA in routine practice. We assessed whether immunological trajectories differed across prespecified subgroups and whether baseline immune status, duration of pre-switch suppression, or post-switch viremia was independently associated with CD4/CD8 ratio change.

## METHODS

### Study Design

The RELATIVITY cohort is an ongoing nationwide, multicenter, ambispective observational cohort study conducted in Spain that includes PWH who switched from oral ART to CAB+RPV LA in routine clinical practice. This updated analysis included data collected through 1 March 2026, from 53 centers. The ambispective design combined the retrospective inclusion of individuals already receiving CAB+RPV LA at cohort initiation with the prospective follow-up of new initiations. Clinical management, HIV-1 RNA monitoring, immune testing, and injection logistics followed local practices; therefore, the analysis describes immune trajectories after switching and does not estimate a causal treatment effect. To reduce bias related to incomplete site updates, 5 centers without fully updated data relative to previously published datasets were excluded [19]. The RELATIVITY cohort was registered at ClinicalTrials.gov (NCT07703748).

### Study Population

Eligible participants were adults (≥18 years) with HIV-1 infection, HIV-1 RNA <50 copies/mL at the time of switch, and at least one intramuscular CAB+RPV LA dose administered before 31 December 2024. Clinical eligibility for CAB+RPV LA followed routine practice and excluded pregnancy, active hepatitis B (HBsAg positive) without concomitant active treatment, prior CAB+RPV LA clinical trial participation, and resistance-associated mutations expected to compromise rilpivirine or second-generation integrase inhibitor activity; K103N alone was allowed [20]. For this analysis, participants with HIV-1 RNA ≥50 copies/mL at the time of switching or no evaluable baseline or follow-up cohort data were excluded.

The prespecified covariates were age (<60 or ≥60 years), sex, recorded geographic origin (Spain, Latin America, Africa, or other regions), comorbidity burden (0–1, 2–3, or ≥4 for subgroup analyses; <2 or ≥2 for modeling), baseline CD4/CD8 ratio (<1.0 or ≥1.0), baseline CD4+ count (<500 or ≥500 cells/mm^3^), CD4+ nadir (<200 or ≥200 cells/mm^3^), prior AIDS, previous virological failure, and ART class in the regimen immediately preceding CAB+RPV LA (an integrase strand transfer inhibitor [INSTI]-, non-nucleoside reverse transcriptase inhibitor [NNRTI]-, or protease inhibitor [PI]-containing regimen). The 1.0 CD4/CD8 ratio threshold was selected a priori for immune profile analyses because it represents ratio normalization and is widely used in treated HIV cohorts [6, 21, 22].

### Data Collection

Data were recorded in a standardized REDCap electronic case report form [23]. Variables included demographics, HIV history, recorded duration of virological suppression before the switch, prior AIDS and virological failure, the ART regimen immediately preceding CAB+RPV LA, and longitudinal CD4+, CD8+, CD4/CD8 ratio, and HIV-1 RNA measurements. Recorded geographic origin combined the nationality field with the conditional origin field: participants recorded as Spanish were categorized as Spain, whereas non-Spanish participants were grouped as Latin America, Africa, or other regions; self-identified ethnicity was unavailable. Comorbidity burden was the recorded count of hypertension, diabetes, dyslipidemia, ischemic heart disease, cerebrovascular disease, peripheral vascular disease, renal impairment, osteopenia/osteoporosis, chronic pulmonary disease, psychiatric disease, active malignancy, alcoholic liver disease, chronic liver disease, nonalcoholic fatty liver disease, active hepatitis C, and active hepatitis B, categorized as 0–1, 2–3, or ≥4.

### Outcomes

The primary immunological outcomes were paired changes from baseline in CD4+ T-cell count, CD8+ T-cell count, and CD4/CD8 ratio. For the nominal 12- and 24-month assessments, measurements from visits within ±1 month were used (months 11 or 13 and months 23 or 25, respectively); when both visits were present, the earlier visit was selected. The nominal 36-month assessment was excluded from the time-point analyses because paired data were available for only 67 participants for CD4+ T-cell count and 65 participants for both CD8+ T-cell count and the CD4/CD8 ratio, yielding estimates that were too imprecise for meaningful interpretation. The last available immune assessment was defined separately for each immune parameter as the most recent available post-baseline measurement. For paired-ratio analyses, time to the last ratio assessment was calculated from recorded assessment and switch dates when available, with the nominal visit month recorded in the electronic case report form used as a fallback. CD4/CD8 ratios were reconstructed from contemporaneous CD4+ and CD8+ counts when available. Values outside 0.01–6.0 were reviewed against component counts and neighboring visits and set to missing only when zero or anomalously low CD8+ values indicated a probable data-entry or export error; 54 ratio measurements were excluded.

Secondary longitudinal virological status was evaluated through each immune assessment. All recorded suppression required at least one post-switch HIV-1 RNA measurement, and all recorded values <50 copies/mL; any viremia was defined as at least one recorded value ≥50 copies/mL. Protocol-defined virological failure used the centrally adjudicated endpoint from the general RELATIVITY analysis: 2 consecutive HIV-1 RNA measurements ≥200 copies/mL or 1 value >500 copies/mL leading to CAB+RPV LA discontinuation. All suspected rebounds were centrally reviewed; a discontinuation-reason flag alone was not sufficient for classification.

### Statistical Analysis

Continuous variables are presented as medians and interquartile ranges (IQRs), and categorical variables as n/N (%). Paired analyses used observed data for participants with baseline and follow-up measurements; no imputation was performed. Within-person changes were tested with Wilcoxon signed-rank tests. Baseline characteristics of participants with and without paired 12-month CD4+ measurements were compared using Wilcoxon rank-sum, χ^2^, or Fisher exact tests. The primary multivariable linear model of CD4/CD8 ratio change at the last available assessment used heteroscedasticity-consistent (HC3) standard errors and included age, sex, recorded geographic origin, ≥2 comorbidities, baseline CD4/CD8 <1.0, baseline CD4+ <500 cells/mm^3^, CD4+ nadir <200 cells/mm^3^, prior AIDS, previous virological failure, immediate pre-switch ART classes, years on ART, duration of pre-switch suppression, any post-switch HIV-1 RNA ≥50 copies/mL, and time to the last ratio assessment. Complete cases were analyzed. Sensitivity analyses were restricted to participants with all recorded post-switch HIV-1 RNA <50 copies/mL and separately excluded protocol-defined virological failures. Subgroup analyses were exploratory and were not adjusted for multiplicity. Two-sided *P* values <.05 were considered statistically significant; effect sizes and confidence intervals guided interpretation. Statistical analyses were conducted using R software (version 4.4.0).

### Ethics

The study protocol was approved by the Ethics Committee for Research with Medicines of the Burgos and Soria Health Area (reference code: 23-00144). All participants provided written informed consent. Data were managed in accordance with applicable data protection regulations and ethical standards for biomedical research.

## RESULTS

### Study Population and Baseline Immune Profile

A total of 3349 participants were included in the study. Baseline CD4/CD8 ratio was available for 2995 participants: 1549 had a CD4/CD8 ratio <1.0, and 1446 had a ratio ≥1.0. The median baseline CD4+ T-cell count was 797 cells/mm^3^ (IQR, 602–1026), CD8+ T-cell count was 826 cells/mm^3^ (IQR, 607–1104), and CD4/CD8 ratio was 0.98 (IQR, 0.70–1.31). The median duration of pre-switch virological suppression was 81 months (IQR, 34–132). Baseline characteristics are shown in Table 1. Baseline CD8+ counts did not differ significantly by recorded geographic origin (Spain, 831 [IQR, 603–1117]; Latin America, 818 [IQR, 618–1059]; Africa, 745 [IQR, 597–1031]; other regions, 796 [IQR, 598–1044] cells/mm^3^; global *P* = .237).

**Table 1.** Baseline Characteristics According to Baseline CD4/CD8 Ratio Category

Variable	Overall	Baseline CD4/CD8 < 1.0	Baseline CD4/CD8 ≥ 1.0	*P* value
Participants, n	2995	1549	1446	
Age, years	45 [37 to 54]	45 [37 to 55]	45 [38 to 54]	.873
Age ≥60 years	371/2995 (12.4)	207/1549 (13.4)	164/1446 (11.3)	.093
Female sex	450/2964 (15.2)	185/1534 (12.1)	265/1430 (18.5)	<.001
Recorded geographic origin: Spain	2121/2974 (71.3)	1075/1538 (69.9)	1046/1436 (72.8)	.107
Recorded geographic origin: Latin America	662/2974 (22.3)	367/1538 (23.9)	295/1436 (20.5)	
Recorded geographic origin: Africa	66/2974 (2.2)	37/1538 (2.4)	29/1436 (2.0)	
Recorded geographic origin: Other regions	125/2974 (4.2)	59/1538 (3.8)	66/1436 (4.6)	
Body mass index, kg/m^2^	25.4 [23.3 to 28.1]	25.4 [23.3 to 28.0]	25.4 [23.2 to 28.3]	.906
Number of comorbidities	0 [0 to 1]	0 [0 to 1]	0 [0 to 1]	.226
≥2 comorbidities	537/2995 (17.9)	287/1549 (18.5)	250/1446 (17.3)	.377
CD4+ T-cell nadir, cells/mm^3^	338 [191 to 496]	270 [134 to 422]	403 [266 to 559]	<.001
CD4+ nadir <200 cells/mm^3^	625/2393 (26.1)	453/1247 (36.3)	172/1146 (15.0)	<.001
Prior AIDS	376/2720 (13.8)	262/1414 (18.5)	114/1306 (8.7)	<.001
Previous virological failure	103/2412 (4.3)	66/1245 (5.3)	37/1167 (3.2)	.010
Baseline CD4+ T-cell count, cells/mm^3^	797 [602 to 1026]	683 [514 to 870]	919 [718 to 1162]	<.001
Baseline CD4+ T-cell count <500 cells/mm^3^	438/2995 (14.6)	364/1549 (23.5)	74/1446 (5.1)	<.001
Baseline CD4+ T-cell count ≥500 cells/mm^3^	2557/2995 (85.4)	1185/1549 (76.5)	1372/1446 (94.9)	<.001
Baseline CD8+ T-cell count, cells/mm^3^	826 [607 to 1104]	1010 [780 to 1308]	662 [506 to 851]	<.001
Baseline CD4/CD8 ratio	0.98 [0.70 to 1.31]	0.71 [0.55 to 0.85]	1.32 [1.14 to 1.65]	<.001
Virological suppression before switch, months	81 [34 to 132]	69 [24 to 122]	93 [48 to 140]	<.001
Years on ART before CAB+RPV LA	9.2 [5.0 to 15.0]	9.0 [4.0 to 15.0]	10.0 [6.0 to 15.0]	<.001
Pre-switch INSTI-containing regimen	2398/2995 (80.1)	1233/1549 (79.6)	1165/1446 (80.6)	.508
Pre-switch NNRTI-containing regimen	966/2995 (32.3)	500/1549 (32.3)	466/1446 (32.2)	.976
Pre-switch PI-containing regimen	183/2995 (6.1)	103/1549 (6.6)	80/1446 (5.5)	.202

Data are median [IQR] or n/N (%). Baseline CD4/CD8 groups (<1.0 vs ≥1.0) were compared using Wilcoxon, χ^2^, or Fisher exact tests. Prior ART denotes the immediate pre-switch regimen; classes may overlap. Geographic origin was recorded and was not a self-identified ethnicity measure.

### Overall Paired Immunological Changes

Paired immunological changes are summarized in Table 2 and Figure 1. In the overall cohort of 3349 participants, median clinical follow-up was 25.1 months (IQR, 19.3–30.8), whereas the median time to the last paired-ratio assessment was 22 months (IQR, 16–27; n = 2929); 394/2929 participants (13.5%) had less than 12 months between switching and that assessment. CD4+ T-cell counts showed no meaningful change over the observation period. At the last available immune assessment, median changes were −7 cells/mm^3^ (IQR, −138 to +117; n = 2992) for CD4+ T-cell count, −60 cells/mm^3^ (IQR, −214 to +75; n = 2941) for CD8+ T-cell count, and +0.07 (IQR, −0.05 to +0.20; n = 2929) for the CD4/CD8 ratio.

**Figure 1.**
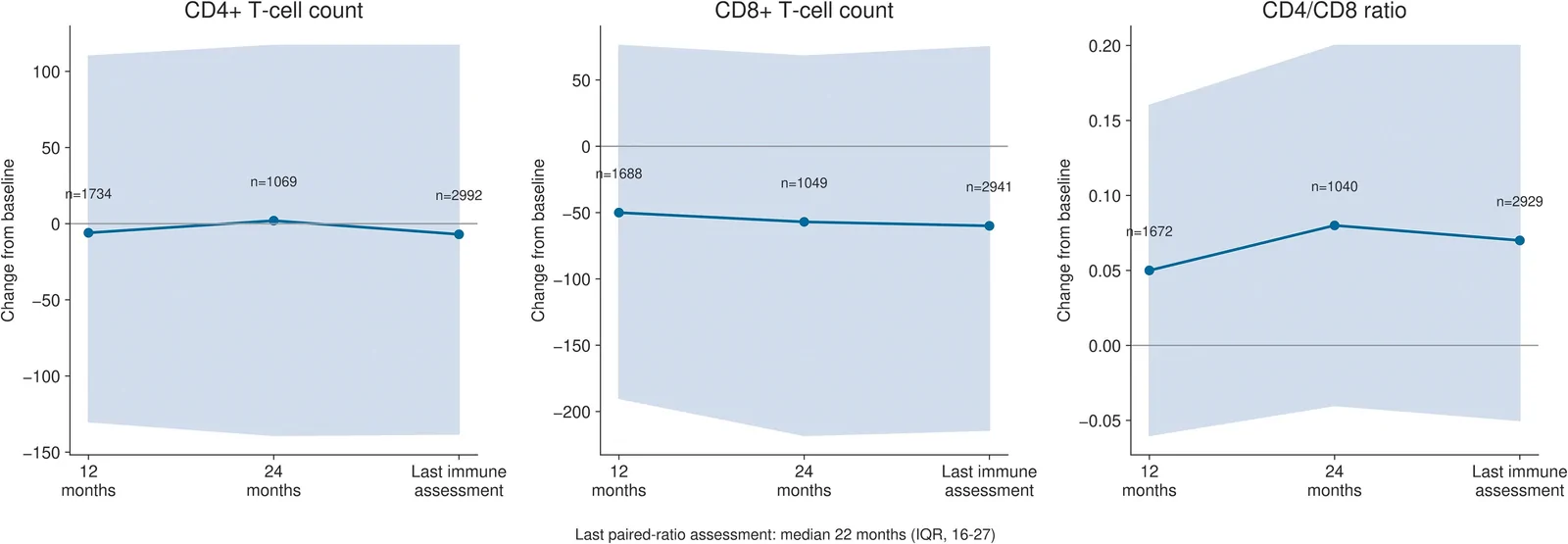
Paired immunological changes after switching to CAB+RPV LA. Points and lines show median within-person change from baseline; shaded areas show IQRs. The 12- and 24-month landmarks used visits within ±1 month, and the last available immune assessment was parameter-specific. Median time to the last paired-ratio assessment was 22 months (IQR, 16–27), calculated from recorded dates with nominal-event fallback. Denominators are displayed at each time point. CAB+RPV LA, long-acting cabotegravir plus rilpivirine; IQR, interquartile range.

**Table 2.** Paired Immunological Changes After Switching to CAB+RPV LA

Time point	Parameter	Paired n	Baseline Median [IQR]	Follow-up Median [IQR]	Median Change [IQR]	Within-Person *P* value
∼12 months	CD4+ T-cell count	1734	795 [604 to 1033]	787 [606 to 992]	−6 [−130 to 110]	.058
	CD8+ T-cell count	1688	833 [619 to 1107]	777 [573 to 1032]	−50 [−190 to 76]	<.001
	CD4/CD8 ratio	1672	0.96 [0.70 to 1.29]	1.02 [0.74 to 1.36]	0.05 [−0.06 to 0.16]	<.001
∼24 months	CD4+ T-cell count	1069	796 [604 to 1036]	773 [601 to 995]	2 [−139 to 117]	.146
	CD8+ T-cell count	1049	818 [606 to 1112]	744 [551 to 1014]	−57 [−218 to 68]	<.001
	CD4/CD8 ratio	1040	0.97 [0.71 to 1.29]	1.05 [0.77 to 1.40]	0.08 [−0.04 to 0.20]	<.001
Last available assessment	CD4+ T-cell count	2992	797 [601 to 1028]	770 [594 to 999]	−7 [−138 to 117]	.004
	CD8+ T-cell count	2941	826 [606 to 1104]	753 [551 to 1012]	−60 [−214 to 75]	<.001
	CD4/CD8 ratio	2929	0.97 [0.70 to 1.31]	1.05 [0.76 to 1.39]	0.07 [−.05 to 0.20]	<.001

Data are medians [IQR]. The ∼12- and ∼24-month assessments used ±1-month windows; last assessments were parameter-specific (median paired-ratio timing, 22 months [IQR, 16–27]). *P* values are from Wilcoxon signed-rank tests. CAB+RPV LA, long-acting cabotegravir plus rilpivirine; IQR, interquartile range.

At the 12-month landmark, 1734/3349 participants (51.8%) had paired CD4+ T-cell count data available. Baseline CD4+ and CD8+ T-cell counts, CD4/CD8 ratio, ART duration, and pre-switch suppression duration did not differ significantly between participants with and without paired CD4+ data. Women and participants from outside Spain were modestly overrepresented among those without paired data. HIV-1 RNA availability through month 13 was 99.9% and 97.0%, respectively (Supplementary Table 1).

Among participants with a paired CD4/CD8 ratio at the last available assessment, 2921/2929 (99.7%) had at least one post-switch HIV-1 RNA result; 2570/2921 (88.0%) had all recorded values <50 copies/mL, and 351/2921 (12.0%) had at least one value ≥50 copies/mL. At the selected immune visit, 2666/2761 (96.6%) with available contemporaneous HIV-1 RNA were suppressed. Protocol-defined virological failure occurred in 28/3349 participants (0.8%) during full follow-up; 3/2929 participants (0.1%) had experienced the adjudicated endpoint by their selected last-ratio assessment (Supplementary Table 2).

### Subgroup Analyses

Changes at the last available assessment across subgroups are shown in Table 3. The largest difference was by baseline immune profile: participants with baseline CD4/CD8 <1.0 had a median ratio increase of +0.09 (IQR, −0.01 to +0.20), compared with +0.05 (IQR, −0.12 to +0.21) among those with baseline CD4/CD8 ≥1.0. Changes by recorded geographic origin were small; median increases were +0.06 for Spain and +0.10 for Latin America, Africa, and other regions.

**Table 3.** Immunological Changes at the Last Available Assessment by Subgroup

Subgroup	Category	n CD4	Delta CD4 Median [IQR]	n CD8	Delta CD8 Median [IQR]	n Ratio	Delta CD4/CD8 Median [IQR]
Age group	<60 y	2625	−7 [−140 to 120]	2576	−63 [−218 to 75]	2566	0.08 [−0.05 to 0.21]
	≥60 y	367	−5 [−132 to 100]	365	−30 [−198 to 74]	363	0.06 [−0.07 to 0.17]
Sex	Male	2521	−8 [−137 to 115]	2474	−60 [−220 to 78]	2464	0.07 [−0.06 to 0.20]
	Female	439	−3 [−143 to 118]	435	−60 [−200 to 50]	434	0.10 [−0.05 to 0.25]
Recorded geographic origin	Spain	2124	−8 [−136 to 112]	2091	−54 [−210 to 78]	2084	0.06 [−0.06 to 0.19]
	Latin America	661	−4 [−147 to 124]	644	−82 [−240 to 59]	640	0.10 [−0.03 to 0.23]
	Africa	65	−15 [−191 to 134]	65	−80 [−211 to 35]	65	0.10 [−0.05 to 0.22]
	Other regions	122	16 [−124 to 136]	121	−54 [−217 to 82]	120	0.10 [−0.03 to 0.22]
Comorbidity burden	0–1	2467	−8 [−134 to 120]	2423	−63 [−218 to 75]	2412	0.07 [−0.05 to 0.21]
	2–3	453	−7 [−158 to 96]	446	−52 [−201 to 71]	445	0.06 [−0.06 to 0.19]
	≥4	72	20 [−108 to 131]	72	−40 [−240 to 64]	72	0.07 [0.01 to 0.14]
Baseline CD4/CD8 ratio	<1.0	1515	12 [−112 to 124]	1513	−99 [−290 to 70]	1509	0.09 [−0.01 to 0.20]
	≥1.0	1419	−27 [−176 to 110]	1417	−35 [−147 to 75]	1420	0.05 [−0.12 to 0.21]
Baseline CD4+ count	<500 cells/mm^3^	440	75 [0 to 156]	432	19 [−141 to 140]	429	0.08 [0.00 to 0.20]
	≥500 cells/mm^3^	2552	−30 [−164 to 105]	2509	−72 [−225 to 55]	2500	0.07 [−0.06 to 0.20]
CD4+ nadir	<200 cells/mm^3^	620	17 [−82 to 109]	610	−49 [−204 to 85]	610	0.07 [−0.02 to 0.18]
	≥200 cells/mm^3^	1787	−20 [−156 to 120]	1751	−68 [−224 to 68]	1740	0.08 [−0.07 to 0.21]
Pre-switch ART class	INSTI	2390	−11 [−144 to 116]	2353	−66 [−224 to 66]	2344	0.08 [−0.05 to 0.21]
	NNRTI	966	3 [−126 to 130]	950	−30 [−196 to 92]	946	0.05 [−0.08 to 0.18]
	PI	186	12 [−123 to 110]	182	−78 [−204 to 88]	181	0.10 [−0.03 to 0.19]
Post-switch virological status	All recorded HIV-1 RNA <50 copies/mL	2605	−4 [−132 to 115]	2581	−59 [−211 to 75]	2570	0.07 [−0.05 to 0.20]
	Any recorded HIV-1 RNA ≥50 copies/mL	354	−22 [−167 to 133]	351	−76 [−246 to 70]	351	0.07 [−0.08 to 0.21]

Data are median changes from baseline [IQR]. Prior ART refers to the immediate pre-switch regimen; classes were not mutually exclusive. Origin categories were Spain, Latin America, Africa, and other regions. Post-switch virological status was assessed through each parameter-specific last immune assessment among participants with at least one post-switch HIV-1 RNA result. ART, antiretroviral therapy; INSTI, integrase strand transfer inhibitor; NNRTI, nonnucleoside reverse transcriptase inhibitor; PI, protease inhibitor.

Participants with all recorded post-switch HIV-1 RNA <50 copies/mL had a median ratio increase of +0.07 (IQR, −0.05 to +0.20), as did those with any recorded HIV-1 RNA ≥50 copies/mL (+0.07 [IQR, −0.08 to +0.21]) (Table 3). The adjusted analysis accounted for baseline and follow-up factors (Supplementary Table 3).

### CD4/CD8 Ratio Categories and Predictors of Ratio Change

Among 2929 participants with paired ratios at baseline and the last available assessment, 323/1509 (21.4%) with a baseline ratio <1.0 reached ≥1.0, whereas 146/1420 (10.3%) with a baseline ratio ≥1.0 moved to <1.0. Consequently, the proportion with a ratio ≥1.0 increased from 1420/2929 (48.5%) to 1597/2929 (54.5%).

The primary complete-case model included 1727 participants (Figure 2 and Supplementary Table 3). Baseline CD4/CD8 <1.0 was associated with a greater ratio increase (adjusted beta, +0.060; 95% CI, +0.030 to +0.090; *P* < .001). Longer pre-switch suppression per 5 years (adjusted beta, −0.028; 95% CI, −.045 to −.012; *P* < .001) and any post-switch HIV-1 RNA ≥50 copies/mL (adjusted beta, −0.054; 95% CI, −.098 to −.010; *P* = .017) were associated with smaller increases. Female sex had a positive association, whereas African origin did not differ independently from Spanish origin. None of the ART classes in the regimen immediately preceding CAB+RPV LA was independently associated with ratio change.

**Figure 2.**
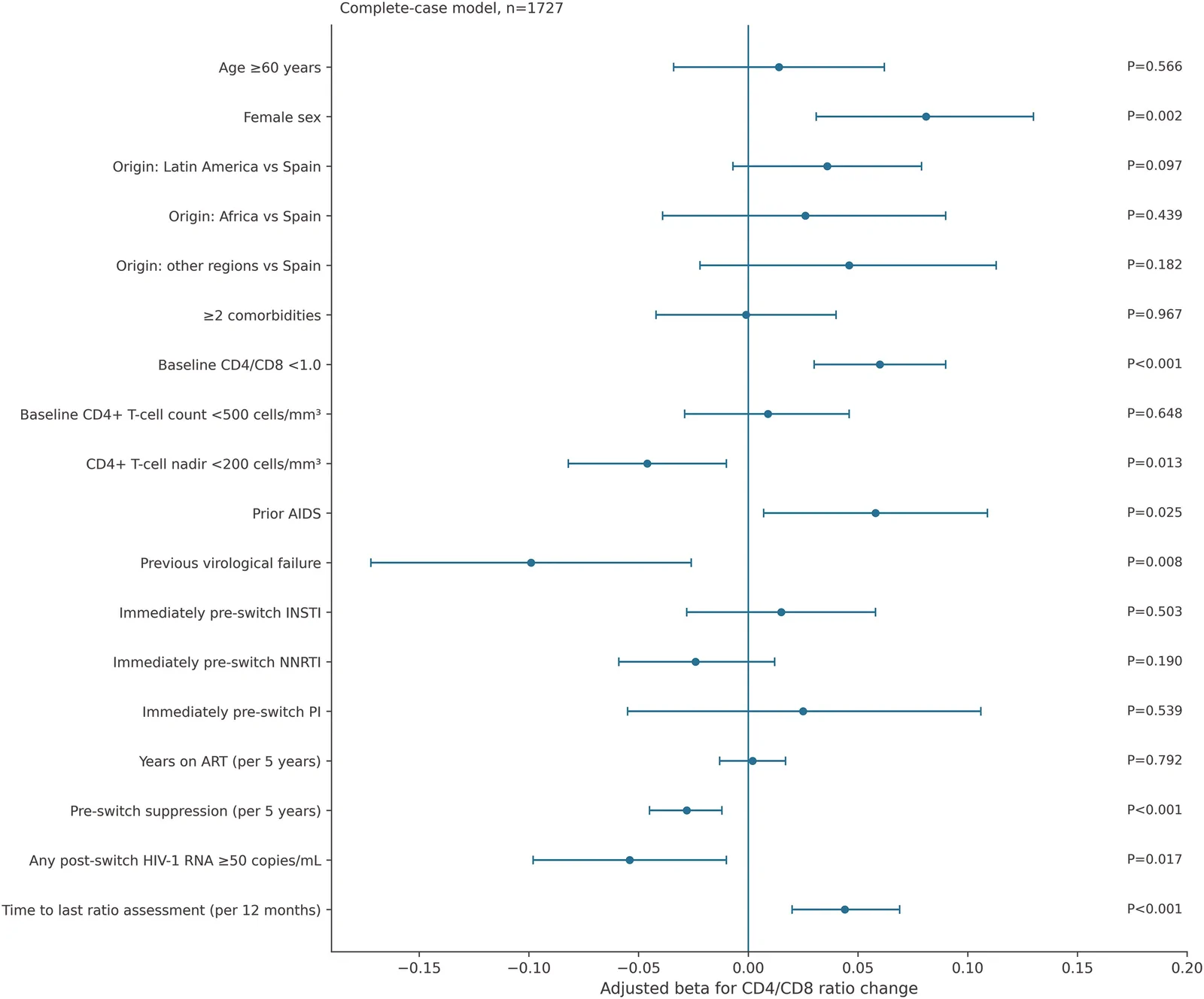
Multivariable correlates of CD4/CD8 ratio change at the last available assessment. Dots are adjusted beta coefficients, and horizontal lines are 95% confidence intervals from the complete-case linear model (n = 1727) using HC3 robust standard errors. ART, antiretroviral therapy; CI, confidence interval; INSTI, integrase strand transfer inhibitor; NNRTI, nonnucleoside reverse transcriptase inhibitor; PI, protease inhibitor.

The association for baseline CD4/CD8 <1.0 persisted among participants with all recorded post-switch HIV-1 RNA <50 copies/mL (n = 1519; adjusted beta, +0.057; 95% CI, +.026 to +.089; *P* < .001) and after excluding 28 protocol-defined virological failures (n = 1712; adjusted beta, +0.061; 95% CI, +.031 to +.092; *P* < .001) (Supplementary Table 4).

## DISCUSSION

In this nationwide real-world cohort of virologically suppressed PWH who switched to CAB+RPV LA, CD4+ T-cell counts showed no meaningful change, while CD8+ T-cell counts declined modestly and the CD4/CD8 ratio increased slightly. The baseline-ratio association was similar when analyses were restricted to participants with all recorded post-switch HIV-1 RNA values <50 copies/mL and when protocol-defined virological failures were excluded. These findings are reassuring regarding immunological stability during effective maintenance therapy, but they remain descriptive and do not establish a CAB+RPV-specific effect.

The distinction between stable CD4+ T-cell counts and modestly declining CD8+ T-cell counts is relevant to interpretation. Expanded CD8+ T-cell populations and a low CD4/CD8 ratio can persist despite suppressive ART [2–8]. The observed median ratio increase of 0.07 was small and descriptive; without an oral ART comparator, it may reflect continued immune evolution during sustained suppression, cohort selection, or measurement timing rather than an effect of CAB+RPV LA itself.

Baseline CD4/CD8 <1.0 was independently associated with a larger increase. This threshold corresponds to the conventional definition of ratio normalization used in treated HIV cohorts [6, 21, 22]. The low-ratio group had greater baseline CD8+ T-cell expansion and a larger subsequent CD8+ T-cell count decline, consistent with more room for change [11, 24]. Because change was defined from the same baseline measure used to form the strata, regression to the mean and mathematical coupling probably contributed; the adjusted beta should not be interpreted as a causal effect of a low baseline ratio.

Female sex was also independently associated with a larger CD4/CD8 ratio increase, although women constituted a minority and the finding is hypothesis-generating. Sex-related differences in immune activation and reconstitution have been reported previously [25–27]. Baseline CD8+ T-cell counts did not differ significantly across recorded-origin groups, and African origin was not independently associated with the ratio change. The available data therefore do not support African origin as an explanation for the sex association. Recorded geographic origin is not equivalent to self-identified ethnicity, and residual confounding remains possible.

Longer pre-switch suppression was associated with smaller ratio increases, compatible with participants approaching an immunological steady state before CAB+RPV LA; conversely, those with shorter prior suppression retained more room for improvement. Any recorded post-switch HIV-1 RNA ≥50 copies/mL was also independently associated with a smaller adjusted increase despite overlapping unadjusted medians. These associations fit a general relationship between durable virological control and gradual immune evolution described across suppressive ART cohorts [6, 11, 21, 22, 28, 29], but individual detectable values may represent blips, and the observational data cannot identify adherence- or antigen-driven mechanisms.

The sensitivity analyses restricted to participants with all recorded post-switch HIV-1 RNA values <50 copies/mL and excluding 28 protocol-defined virological failures reproduced the baseline-ratio association. Immediate pre-switch INSTI, NNRTI, and PI exposure was not independently associated with ratio change. These analyses reduce concern that the main association was driven by virological failure or the immediately preceding ART class, but they do not eliminate residual confounding.

Our results complement those of trials and real-world studies establishing the virological efficacy and durability of CAB+RPV LA [12–19]. Smaller cohorts have also reported stable CD4+ T-cell counts and increasing CD4/CD8 ratios after switching [27, 30, 31]. RELATIVITY adds paired CD8+ T-cell count data, a large national sample, and explicit accounting for pre-switch suppression duration and longitudinal viremia while remaining limited by the absence of an oral ART comparator.

The clinical implications are modest. Current guidance makes CD4+ T-cell count monitoring optional in people with durable suppression and adequate counts and advises against routine monitoring of lymphocyte subsets other than CD4+ T-cells [32]. Accordingly, these data do not support additional routine CD8+ or CD4/CD8-ratio monitoring after a switch to CAB+RPV LA. These measures may remain useful research outcomes or selected clinical descriptors, but this study did not test monitoring strategies or predict clinical events.

Limitations include the ambispective observational design, selection and residual confounding, exclusion of 5 centers with incomplete updates, and absence of an oral-ART control group. Immune and HIV-1 RNA testing followed routine practice, producing variable denominators and a parameter-specific last-available-assessment endpoint that combined different observation times; 13.5% of paired-ratio assessments occurred before 12 months, although time to the last ratio assessment was reported and included in the model. Only 51.8% of the cohort had paired 12-month CD4+ T-cell count data, and the complete-case model included 1727 participants; measured baseline immune characteristics were similar by 12-month CD4+ availability, but selection on unmeasured factors remains possible. HIV-1 RNA sampling could miss transient viremia, serial pre-switch HIV-1 RNA trajectories were not collected uniformly, pre-switch suppression duration was site-reported, and virological failure was uncommon. Failure analyses used the centrally adjudicated definition shared with the general RELATIVITY analysis rather than an unverified discontinuation-reason flag. Recorded geographic origin combined Spanish nationality with a conditional origin field for non-Spanish participants and should not be interpreted as self-identified ethnicity. Regression to the mean and mathematical coupling may have contributed to the baseline-group association. A total of 54 CD4/CD8 ratio measurements linked to internally inconsistent component counts were excluded after source-data review. Subgroup analyses were exploratory and not adjusted for multiplicity. We lacked inflammatory biomarkers, T-cell activation phenotypes, cytomegalovirus serostatus, pre-switch adherence measures, and power for clinical events. Statistical significance in this large cohort should not be equated with clinical importance.

## CONCLUSIONS

Among virologically suppressed PWH receiving CAB+RPV LA, CD4+ T-cell counts showed no meaningful change, CD8+ T-cell counts declined modestly, and the CD4/CD8 ratio increased slightly. In this selected cohort, these findings are consistent with overall immunological stability; the small ratio increase primarily reflected CD8+ decline and should not be interpreted as a CAB+RPV LA-specific effect.

## Supplementary Material

ofag583_Supplementary_Data

## Supplementary Data

Supplementary materials are available at *Open Forum Infectious Diseases* online. Consisting of data provided by the authors to benefit the reader, the posted materials are not copyedited and are the sole responsibility of the authors, so questions or comments should be addressed to the corresponding author.

## References

[1] Deeks SG, Lewin SR, Havlir DV. The end of AIDS: HIV infection as a chronic disease. Lancet 2013; 382:1525–33. 10.1016/S0140-6736(13)61809-7 PMC4058441 24152939

[2] Hunt PW, Lee SA, Siedner MJ. Immunologic biomarkers, morbidity, and mortality in treated HIV infection. J Infect Dis 2016; 214:S44–50. 10.1093/infdis/jiw275 PMC5021241 27625430

[3] Tenorio AR, Zheng Y, Bosch RJ, et al Soluble markers of inflammation and coagulation but not T-cell activation predict non-AIDS-defining morbid events during suppressive antiretroviral treatment. J Infect Dis 2014; 210:1248–59. 10.1093/infdis/jiu254 PMC4192039 24795473

[4] Serrano-Villar S, Pérez-Elías MJ, Dronda F, et al Increased risk of serious non-AIDS-related events in HIV-infected subjects on antiretroviral therapy associated with a low CD4/CD8 ratio. PLoS One 2014; 9:e85798. 10.1371/journal.pone.0085798 PMC3907380 24497929

[5] Serrano-Villar S, Sainz T, Lee SA, et al HIV-infected individuals with low CD4/CD8 ratio despite effective antiretroviral therapy exhibit altered T-cell subsets, heightened CD8+ T-cell activation, and increased risk of non-AIDS morbidity and mortality. PLoS Pathog 2014; 10:e1004078. 10.1371/journal.ppat.1004078 PMC4022662 24831517

[6] Mussini C, Lorenzini P, Cozzi-Lepri A, et al CD4/CD8 ratio normalisation and non-AIDS-related events in individuals with HIV who achieve viral load suppression with antiretroviral therapy: an observational cohort study. Lancet HIV 2015; 2:e98–106. 10.1016/S2352-3018(15)00006-5 26424550

[7] Serrano-Villar S, Wu K, Hunt PW, et al Predictive value of CD8+ T cell and CD4/CD8 ratio at two years of successful ART in the risk of AIDS and non-AIDS events. EBioMedicine 2022; 80:104072. 10.1016/j.ebiom.2022.104072 PMC9156990 35644125

[8] Martínez-Sanz J, Díaz-Álvarez J, Rosas M, et al Expanding HIV clinical monitoring: the role of CD4, CD8, and CD4/CD8 ratio in predicting non-AIDS events. EBioMedicine 2023; 95:104773. 10.1016/j.ebiom.2023.104773 PMC10474064 37639938

[9] Trickey A, May MT, Schommers P, et al CD4:CD8 ratio and CD8 count as prognostic markers for mortality in human immunodeficiency virus-infected patients on antiretroviral therapy: the Antiretroviral Therapy Cohort Collaboration (ART-CC). Clin Infect Dis 2017; 65:959–66. 10.1093/cid/cix466 PMC5850630 28903507

[10] Ron R, Martínez-Sanz J, Herrera S, et al CD4/CD8 ratio and CD8+ T-cell count as prognostic markers for non-AIDS mortality in people living with HIV: a systematic review and meta-analysis. Front Immunol 2024; 15:1343124. 10.3389/fimmu.2024.1343124 PMC10868578 38361925

[11] Serrano-Villar S, Martínez-Sanz J, Ron R, et al Effects of first-line antiretroviral therapy on the CD4/CD8 ratio and CD8 cell counts in CoRIS: a prospective multicentre cohort study. Lancet HIV 2020; 7:e565–73. 10.1016/S2352-3018(20)30202-2 32763219

[12] Swindells S, Andrade-Villanueva JF, Richmond GJ, et al Long-acting cabotegravir and rilpivirine for maintenance of HIV-1 suppression. N Engl J Med 2020; 382:1112–23. 10.1056/NEJMoa1904398 32130809

[13] Orkin C, Arasteh K, Górgolas Hernández-Mora M, et al Long-acting cabotegravir and rilpivirine after oral induction for HIV-1 infection. N Engl J Med 2020; 382:1124–35. 10.1056/NEJMoa1909512 32130806

[14] Rizzardini G, Overton ET, Orkin C, et al Long-acting injectable cabotegravir + rilpivirine for HIV maintenance therapy: week 48 pooled analysis of phase 3 ATLAS and FLAIR trials. J Acquir Immune Defic Syndr 2020; 85:498–506. 10.1097/QAI.0000000000002466 PMC7592884 33136751

[15] Overton ET, Richmond G, Rizzardini G, et al Long-acting cabotegravir and rilpivirine dosed every 2 months in adults with human immunodeficiency virus 1 type 1 infection: 152-week results from ATLAS-2M, a randomized, open-label, phase 3b, noninferiority study. Clin Infect Dis 2023; 76:1646–54. 10.1093/cid/ciad020 PMC10156123 36660819

[16] Ramgopal MN, Castagna A, Cazanave C, et al Efficacy, safety, and tolerability of switching to long-acting cabotegravir plus rilpivirine versus continuing fixed-dose bictegravir, emtricitabine, and tenofovir alafenamide in virologically suppressed adults with HIV, 12-month results (SOLAR): a randomised, open-label, phase 3b, non-inferiority trial. Lancet HIV 2023; 10:e566–77. 10.1016/S2352-3018(23)00136-4 37567205

[17] Kityo C, Mambule IK, Musaazi J, et al Cabotegravir and rilpivirine for treatment of HIV infection in Africa: week 96 results from the phase 3b randomized, open-label, noninferiority CARES trial. Nat Med 2026; 32:168–77. 10.1038/s41591-025-04041-7 PMC12823427 41188524

[18] Sension MG, Brunet L, Hsu RK, et al Cabotegravir + rilpivirine long-acting injections for HIV treatment in the US: real world data from the OPERA cohort. Infect Dis Ther 2023; 12:2807–17. 10.1007/s40121-023-00890-2 PMC10746614 37966701

[19] Buzón-Martín L, Montes ML, Pedrero R, et al A prospective assessment of the efficacy and durability of long-acting cabotegravir and rilpivirine in individuals with HIV in Spain (RELATIVITY study). J Antimicrob Chemother 2025; 80:3438–51. 10.1093/jac/dkaf389 41128149

[20] Wensing AM, Calvez V, Ceccherini-Silberstein F, et al 2025 update of the drug resistance mutations in HIV-1. Top Antivir Med 2025; 33:457–73. 40472382

[21] Winston A, Jose S, Fisher M, et al Host, disease, and antiretroviral factors are associated with normalization of the CD4:CD8 ratio after initiating antiretroviral therapy. J Allergy Clin Immunol 2015; 136:1682–5.e1. 10.1016/j.jaci.2015.05.047 26253341

[22] Han WM, Apornpong T, Kerr SJ, et al CD4/CD8 ratio normalization rates and low ratio as prognostic marker for non-AIDS defining events among long-term virologically suppressed people living with HIV. AIDS Res Ther 2018; 15:13. 10.1186/s12981-018-0200-4 PMC6158807 30261902

[23] Harris PA, Taylor R, Thielke R, Payne J, Gonzalez N, Conde JG. Research electronic data capture (REDCap)–a metadata-driven methodology and workflow process for providing translational research informatics support. J Biomed Inform 2009; 42:377–81. 10.1016/j.jbi.2008.08.010 PMC2700030 18929686

[24] Cao W, Mehraj V, Trottier B, et al Early initiation rather than prolonged duration of antiretroviral therapy in HIV infection contributes to the normalization of CD8 T-cell counts. Clin Infect Dis 2016; 62:250–7. 10.1093/cid/civ809 PMC4690481 26349551

[25] Maman D, Pujades-Rodriguez M, Subtil F, et al Gender differences in immune reconstitution: a multicentric cohort analysis in sub-Saharan Africa. PLoS One 2012; 7:e31078. 10.1371/journal.pone.0031078 PMC3281917 22363550

[26] Wang X, Xiao J, Zhang L, et al Longitudinal analysis of immune reconstitution and metabolic changes in women living with HIV: a real-world observational study. Chin Med J (Engl) 2023; 136:2168–77. 10.1097/CM9.0000000000002756 PMC10508409 37580056

[27] Spampinato S, Venanzi Rullo E, Conti GN, et al CD4/CD8 ratio increase in female living with HIV switching to cabotegravir-rilpivirine: a real-life 24 weeks evaluation. Pathogens 2025; 14:633. 10.3390/pathogens14070633 PMC12298190 40732681

[28] Castillo-Mancilla JR, Phillips AN, Neaton JD, et al Association of suboptimal antiretroviral therapy adherence with inflammation in virologically suppressed individuals enrolled in the SMART study. Open Forum Infect Dis 2017; 5:ofx275. 10.1093/ofid/ofx275 PMC5772402 29362724

[29] Herrera S, Fernandez-Felix BM, Hunt PW, et al Impact of first-line antiretroviral therapy regimens on the restoration of the CD4/CD8 ratio in the CNICS cohort. J Antimicrob Chemother 2020; 75:1604–10. 10.1093/jac/dkaa024 PMC7225872 32211777

[30] Muccini C, Gianotti N, Lolatto R, Nozza S, Diotallevi S, Castagna A. CD4+/CD8+ improvement after switch from a second-generation integrase inhibitor regimen to long-acting cabotegravir and rilpivirine. AIDS 2024; 38:1890–2. 10.1097/QAD.0000000000003984 39325006

[31] Loi G, Capra N, Lolatto R, et al Increasing CD4/CD8 ratio confirmed after two years of long-acting cabotegravir and rilpivirine in the SCohoLART study. Front Virol 2026; 6:1819287.

[32] Panel on Antiretroviral Guidelines for Adults and Adolescents . Plasma HIV-1 RNA (Viral Load) and CD4 Count Monitoring. Clinicalinfo.HIV.gov. Updated September 25, 2025. Accessed August 24, 2026. Available at: https://clinicalinfo.hiv.gov/en/guidelines/hiv-clinical-guidelines-adult-and-adolescent-arv/plasma-hiv-1-rna-cd4-monitoring.

