# Supplementary material for: Immune Trajectories After Switch to Long-Acting Cabotegravir Plus Rilpivirine in the RELATIVITY Cohort
Source: Open Forum Infect Dis. 2026 Oct 23;13(10):ofag583. doi: 10.1093/ofid/ofag583 (PMC13693702; doi:10.1093/ofid/ofag583)
Supplement: Supplementary file 1 [file ofag583_supplementary_data.docx]

**Supplementary Table S1. Baseline comparison by availability of paired CD4+ data at 12 months**

| **Baseline characteristic** | **Paired CD4 at 12 months (n=1734)** | **No paired CD4 at 12 months (n=1615)** | **P value** |
| --- | --- | --- | --- |
| Age, years | 45 [38 to 54] | 45 [36 to 55] | 0.218 |
| Female sex | 232/1719 (13.5) | 267/1597 (16.7) | 0.010 |
| Recorded geographic origin: Spain | 1241/1721 (72.1) | 1090/1605 (67.9) | 0.025 |
| Recorded geographic origin: Latin America | 390/1721 (22.7) | 399/1605 (24.9) |  |
| Recorded geographic origin: Africa | 28/1721 (1.6) | 40/1605 (2.5) |  |
| Recorded geographic origin: Other regions | 62/1721 (3.6) | 76/1605 (4.7) |  |
| Baseline CD4+ T-cell count, cells/mm³ | 795 [604 to 1033] | 799 [600 to 1020] | 0.931 |
| Baseline CD8+ T-cell count, cells/mm³ | 833 [616 to 1107] | 806 [590 to 1095] | 0.216 |
| Baseline CD4/CD8 ratio | 0.96 [0.70 to 1.29] | 1.00 [0.69 to 1.32] | 0.421 |
| Virological suppression before switch, months | 78 [30 to 130] | 84 [36 to 139] | 0.086 |
| Years on ART before switch | 9 [5 to 15] | 9 [5 to 15] | 0.989 |
| At least one VL recorded through month 13 | 1732/1734 (99.9) | 1567/1615 (97.0) | <0.001 |
| Any HIV-1 RNA ≥50 copies/mL through month 13 among participants with ≥1 recorded VL | 162/1732 (9.4) | 124/1567 (7.9) | 0.142 |

Data are median [IQR] or n/N (%). Overall P values are shown for multi-category variables. The month-13 viremia comparison is restricted to participants with at least one recorded HIV-1 RNA result. VL, viral load.

**Supplementary Table S2. Longitudinal virological status at immune assessment timepoints**

| **Immune assessment** | **Paired ratio n** | **≥1 post-switch VL recorded, n/N (%)** | **All recorded VL <50, n/N with VL (%)** | **Any recorded VL ≥50, n/N with VL (%)** | **VL <50 at selected visit, n/N with VL (%)** | **Protocol-defined VF by assessment, n/N (%)** |
| --- | --- | --- | --- | --- | --- | --- |
| ~12 months | 1672 | 1670/1672 (99.9) | 1513/1670 (90.6) | 157/1670 (9.4) | 1556/1616 (96.3) | 3/1672 (0.2) |
| ~24 months | 1040 | 1040/1040 (100.0) | 907/1040 (87.2) | 133/1040 (12.8) | 955/980 (97.4) | 0/1040 (0.0) |
| Last available assessment | 2929 | 2921/2929 (99.7) | 2570/2921 (88.0) | 351/2921 (12.0) | 2666/2761 (96.6) | 3/2929 (0.1) |

Suppression required ≥1 post-switch HIV-1 RNA result, all <50 copies/mL. Centrally adjudicated VF was counted by assessment date (28 overall); timepoint subsets were nonnested. VF, virological failure; VL, viral load.

**Supplementary Table S3. Multivariable model of CD4/CD8 ratio change**

| **Covariate** | **Adjusted beta** | **95% CI** | **P value** |
| --- | --- | --- | --- |
| Age ≥60 years | +0.014 | -0.034 to +0.062 | 0.566 |
| Female sex | +0.081 | +0.031 to +0.130 | 0.002 |
| Origin: Latin America vs Spain | +0.036 | -0.007 to +0.079 | 0.097 |
| Origin: Africa vs Spain | +0.026 | -0.039 to +0.090 | 0.439 |
| Origin: other regions vs Spain | +0.046 | -0.022 to +0.113 | 0.182 |
| ≥2 comorbidities | -0.001 | -0.042 to +0.040 | 0.967 |
| Baseline CD4/CD8 <1.0 | +0.060 | +0.030 to +0.090 | <0.001 |
| Baseline CD4+ <500 cells/mm³ | +0.009 | -0.029 to +0.046 | 0.648 |
| CD4+ nadir <200 cells/mm³ | -0.046 | -0.082 to -0.010 | 0.013 |
| Prior AIDS | +0.058 | +0.007 to +0.109 | 0.025 |
| Previous virological failure | -0.099 | -0.172 to -0.026 | 0.008 |
| Immediately pre-switch INSTI | +0.015 | -0.028 to +0.058 | 0.503 |
| Immediately pre-switch NNRTI | -0.024 | -0.059 to +0.012 | 0.190 |
| Immediately pre-switch PI | +0.025 | -0.055 to +0.106 | 0.539 |
| Years on ART (per 5 years) | +0.002 | -0.013 to +0.017 | 0.792 |
| Pre-switch suppression (per 5 years) | -0.028 | -0.045 to -0.012 | <0.001 |
| Any post-switch HIV-1 RNA ≥50 copies/mL | -0.054 | -0.098 to -0.010 | 0.017 |
| Time to last ratio assessment (per 12 months) | +0.044 | +0.020 to +0.069 | <0.001 |

Complete-case model (n=1727; HC3 robust standard errors). Positive β indicates a larger increase in the CD4/CD8 ratio; reference groups were the counterparts of those shown. Missing data were excluded.

S**upplementary Table S4. Sensitivity analyses for baseline CD4/CD8 <1.0 and last-assessment ratio change**

| **Analysis** | **Model n** | **Baseline CD4/CD8 <1.0 adjusted beta (95% CI); P** |
| --- | --- | --- |
| Primary model | 1727 | +0.060 (+0.030 to +0.090); P<0.001 |
| Restricted to participants with all recorded post-switch HIV-1 RNA <50 copies/mL | 1519 | +0.057 (+0.026 to +0.089); P<0.001 |
| Excluding protocol-defined virological failures | 1712 | +0.061 (+0.031 to +0.092); P<0.001 |

All models used HC3 robust standard errors and the same covariate specification as the primary model. The failure-exclusion analysis omitted all 28 protocol-defined virological failures. CI, confidence interval.
